# Reliability assessment of the ‘field audit for children’s active transport routes to school’ (FACTS) tool

**DOI:** 10.1186/s12889-024-20285-3

**Published:** 2024-10-14

**Authors:** Thomas V. Vasey, Michael J. Dale, Suzanne J. Carroll

**Affiliations:** Health Research Institute, University of Canberra, Canberra, ACT Australia

**Keywords:** Active school travel, Active school transport, Active school transportation, Safe routes to school, Microscale audit tool, Evaluation, Built environment, Children

## Abstract

**Background:**

Children’s active travel to school is associated with physical activity and thus health. Safe Routes to School (SR2S) programs identify ‘safe routes’ to promote children’s active travel to school. No field audit tool exists specifically to assess the microscale built environment of these ‘safe routes’ within Australia. This study describes the reliability assessment of the Field Audit for Children’s Active Transport to School (FACTS) tool.

**Methods:**

The FACTS tool was developed using a multi-step process, including a literature search, expert opinion, and pilot testing. For the reliability assessment, two trained auditors collected data at eight schools over three weeks in April 2021. For inter-rater reliability, auditors conducted audits on the ‘safe routes’ for the same six schools. For intra-rater reliability, auditors conducted repeat measures on the ‘safe routes’ for four schools each (eight schools total), including three different schools each from the six used for inter-rater reliability and one additional school. Item-by-item reliability was assessed using Cohen’s Kappa, Cohen’s Weighted Kappa, and percentage agreement. The reliability of calculated domain scores was assessed using intraclass correlation coefficients.

**Results:**

For inter-rater reliability, 31 of the 45 (68.9%) items had moderate to almost perfect agreement, seven items (15.6%) had below moderate agreement, and a Kappa statistic could not be calculated for seven items (15.6%) due to constant values. For intra-rater reliability, 37 of the 45 (82.2%) items had moderate to almost perfect agreement, two items (4.4%) had below moderate agreement, and a Kappa statistic could not be calculated for six items (13.3%) due to constant values. For inter- and intra-rater reliability of the segment domain scores, three of the four domains had substantial to almost perfect agreement. For inter- and intra-rater reliability of the crossing domain scores, all four domains had moderate to almost perfect agreement. For inter- and intra-rater reliability of the segment, crossing, route, and school scores, all had substantial to perfect agreement.

**Conclusions:**

The FACTS tool can reliably characterise the microscale built environment of promoted ‘safe routes’ for their use within SR2S programs, and should be considered for use in future SR2S programs within the suburban Australian context.

**Supplementary Information:**

The online version contains supplementary material available at 10.1186/s12889-024-20285-3.

## Introduction

Physical activity is an important behaviour for maintaining health. For children, physical activity is associated with a variety of health benefits, such as improved cardiometabolic health, cardiorespiratory fitness, academic performance, and mental health outcomes [1]. It is therefore recommended that children and youth (5–17 years) participate in at least 60 minutes of moderate to vigorous physical activity daily [2]. Yet, an analysis of physical activity report cards across 57 countries found that only 27 to 33% of children and youth meet this recommendation [3]. Within Australia, it is estimated that only 20 to 26% of children and youth meet this recommendation [3, 4].

Active School Travel (AST), such as walking or cycling to and/or from school, is recognised as a simple and cost-effective strategy for integrating physical activity into children’s daily routines. AST has been associated with increases in daily moderate to vigorous physical activity [5, 6], as well as other physical health benefits [7, 8]. Despite this, many high-income nations have reported significant declines in AST over recent decades [9, 10]. The factors driving the declines in AST are multifaceted, with elements at the intra/inter-personal, family/household, physical/built, and social environmental levels playing important roles in travel mode decision-making [11–13].

To address the ongoing declines in AST, programs such as Safe Routes to School (SR2S) have been implemented across many high-income countries [14–16]. These programs aim to increase children’s engagement in AST by making the neighbourhood environment surrounding primary schools safer to traverse [17]. One of the ways they do this is by identifying, mapping, and clearly marking (e.g., stencils on the footpaths) ‘safe routes’, (i.e., safe travel routes from drop zones to the primary school that avoid main roads and crossings), as well as the promotion of these ‘safe routes’. These routes are also assessed for potential maintenance/infrastructure changes to the built environment (e.g., building of footpaths or placement of a crossing) to improve route quality, safety, and/or convenience [17].

Built environment characteristics include both macroscale features (e.g., dwelling density, intersection density, and land use mix) and microscale features (e.g., footpath quality, signalisation of crossings, and traffic calming infrastructure). Macroscale features are commonly expressed using Geographic Information Systems that draw on a wide range of administrative and/or other datasets [18, 19]. In contrast, microscale features are usually captured through field audits using trained auditors and direct observation [18, 20]. Importantly, microscale features are more likely than macroscale features to influence children’s active travel [21], and they are generally more modifiable [22, 23], making them the primary target of route-level built environmental maintenance/infrastructure changes within SR2S programs. Therefore, assessing route-level microscale features associated with children’s AST is important for informing SR2S program implementation.

Field audit tools are ideal for assessing microscale features that are hypothesised as being associated with active travel. Exemplar field audit tools, such as the Systematic Pedestrian and Cycling Environmental Scan [24], the Pedestrian Environment Data Scan [25], and the Microscale Audit of Pedestrian Streetscapes (MAPS) [20], have been developed to assess characteristics of the built environment at the microscale. These tools are primarily intended to summarise microscale characteristics for a defined area (e.g., ‘neighbourhood’) to express an area ‘walkability’ value. This is often achieved by randomly sampling routes and street segments (e.g., section between two crossings) within a defined area and summating their scores [18, 20, 24, 25]. Overall, field audit tools have been shown to have generally high reliability for assessing the microscale built environment in local contexts, with recent adaptations having demonstrated high reliability internationally [26].

The current suite of field audit tools has several limitations when considering their suitability for assessing the microscale built environment within SR2S programs. Fundamental to these programs are the defined ‘safe routes’. Thus, the microscale built environment of interest and importance within these programs are along these ‘safe routes’, rather than the neighbourhood more broadly. Despite this clear difference in context between SR2S programs and the intended use of other audit tools (i.e., summarising the built environment for a whole neighbourhood), a tool specific for use in Australian SR2S programs remains absent from the literature. Moreover, to ensure their broad application and relevance, previous field audit tools have often been designed to assess the microscale built environment for a range of age groups, including children, youth, adults, and seniors [24–28]. Although many of the tools contain some content relevant to children (e.g., questions to assess cul-de-sac as play areas) [20, 26], they are not designed specifically focused on children and lack the required specificity to adequately assess the microscale built environment for children’s AST within SR2S programs. Furthermore, many of the existing tools include content not relevant to the route-level microscale focus of SR2S programs, such as macroscale questions related to land use and destinations, which can add unnecessary time to data collection and analysis [20, 24, 26]. This is particularly important given that field audits are frequently criticised for their time and resource-intensive nature [18, 19, 28, 29]. Google Street View-based audit tools do exist, which can reduce time and resource burden by allowing desktop-based assessment [19, 28]. However, these tools are unsuitable for use in SR2S programs, as many of the designated ‘safe routes’ use off-street footpaths that lack comprehensive and up to date coverage in Google Street View imagery.

There is need for a reliable and streamlined microscale field audit tool for capturing information on route-level microscale built environment characteristics specific to children’s AST. Such a tool could be used to quantify the quality of key components of the ‘safe routes’, providing path segment- and crossing-based information that can be summarised to the route and school level. Information collected could be used to identify key issues along routes to target for maintenance or infrastructure improvement, as well as to evaluate the effectiveness of SR2S interventions by accurately assessing the environment of interest (i.e., safe routes) at the route and school level. The present study described the reliability assessment of the Field Audit for Children’s Active Transport to School (FACTS) tool and its associated scoring protocol for characterisation of the microscale built environment of promoted SR2S for children’s AST within a suburban Australian environment. Background information on the tool’s development is also provided. This background information is contextual only, and a detailed description of the development of the tool is outside the scope of this paper.

## Methods and materials

### Research context

The reliability assessment of the FACTS tool was conducted within the context of the Active Streets for Schools program [30]. The program, funded and implemented in the suburban areas of the city of Canberra by the Australian Capital Territory Government, is a multi-component SR2S program designed to encourage more children to engage in regular AST by making the environment around schools safer. All schools enrolled on the program received educational materials, a school specific SR2S map outlining their ‘safe routes’, wayfinding stencils and signage, and a single targeted environmental infrastructure change. In total, 81 primary schools enrolled on the program during 2015–2022.

## FACTS tool development (background context)

### Literature search and expert opinion

A search of the literature was performed to identify any relevant microscale audit tools. Twenty-two commonly used validated tools were identified. To assess the relevancy of the identified tools for children’s AST, a panel of five experts in the fields of intervention and evaluation research, GIS/built environment measures and their development, health, and children’s AST/physical activity and behaviour research was convened. These individuals reviewed the content, presentation, and formatting of the identified microscale audit tools. From the tools reviewed, the MAPS Global tool [26] was selected as the base from which to develop the FACTS tool. This decision was driven by (1) the structure of the MAPS Global tool, which replicated our conception of a route (i.e., a predefined path from a drop off point to a destination school) as a series of segments (i.e., a section of the route running either alongside a road, or through a greenway) and crossings (i.e., a section of the route where the path crosses a road – either by overpass, underpass, pedestrian crossing, or a crossing point devoid of formal structure); and (2) the broad applicability of much of the segment and crossing content. The MAPS Global tool’s ‘route level’ and ‘cul-de-sac’ specific components were removed, although certain elements were retained in the ‘segment’ component of the FACTS tool. We then assessed items included in the segment and crossing components for relevance within SR2S programs, and for items considered relevant we: (1) checked/updated the language for the Australian context (e.g., ‘sidewalk’ became ‘path’ and we used metric measures); (2) simplified multiple questions into fewer questions to assist ease of tool use; and (3) simplified response categories (e.g., we considered one obstruction to be of consequence in terms of path safety – making a path potentially unsafe. Capturing data on additional obstructions was not considered useful so the response options were simplified to Yes/No for obstructions rather than None/Some/Many). Lastly, we added in items considered important based on the literature and expert review. Though the FACTS tool primarily drew upon the design and content of the MAPS Global tool, item inclusion and wording were also informed by other identified tools (e.g., the Active Neighborhood Checklist [31]). During the iterative refinement of the FACTS tool, preliminary drafts were shared with the project’s industry partner for sense checking against their real-world knowledge and experience, and to provide input on revisions. A pilot, paper-based version of the FACTS tool consisting of both a segment and crossing component (each purposely designed to fit on one page), was then finalised ready for pilot testing.

### Pilot testing

The FACTS tool was initially pilot tested as part of a field practical performed by undergraduate student Research Assistants (RAs; *n* = 18) who were enrolled in a Public Health course at the University of Canberra. Training consisted of two, two-hour seminars scheduled one week apart, with an in-the-field training exercise conducted between seminars. The initial seminar consisted of a detailed classroom walkthrough of the use of the tool in the field. The student RAs were then assigned two ‘safe routes’ from a local school to audit. These ‘safe routes’ had been audited by one of the authors (MJD) two days prior to the first seminar. The second seminar consisted of a brief revision of tool use and calibration of the student RAs’ audit ratings against the criterion measures (i.e., MJD’s audit ratings). Little variation was identified at this stage, although there were some temporally-dependent variations (e.g., presence and degree of litter and obstructions such as bins). A randomly selected sub-sample of schools (*n* = 27, routes *n* = 152, segments *n* = 805, crossings *n* = 482) enrolled on the Active Streets for Schools program were then audited by the student RAs. The student RAs were assigned two schools each (with some overlap between RAs and schools) and provided with a paper-based version of a prototype FACTS tool, as well as paper-based SR2S maps for their assigned schools, highlighting the ‘safe routes’ to guide the auditing. The student RAs moved in pairs for safety, but each route was audited by one student at a time.

Feedback on the utility of the pilot version of the tool was collected through written reports (*n* = 18) and individual semi-structured interviews with a convenience sample of the student RAs (*n* = 4). All interviews were conducted by one author (TVV). Recruitment for the semi-structured interviews was conducted between 25/06/2019 and 23/07/2019. All interview participants provided informed consent prior to the interviews being conducted. The interviews were conducted to obtain more detailed, open-ended responses than the written reports (the interview question guide has been provided in Additional File 1). The output of the reports and interviews was collated and used to inform further refinement of the FACTS tool. For example, the ordering of several questions was revised to better align with on-the-ground audit progression. Following refinement, the original paper version of the FACTS tool was updated (see Additional File 2). A consistent theme throughout the feedback was the perceived value of digitising the FACTS tool. In response, a digitised version of the tool was developed.

### Digitisation

The FACTS tool was digitised to improve ease of use in the field, and to reduce data entry workload and error rate. Digitisation also allowed for the integration of image capture, geo-tagging for locations (e.g., for litter, graffiti, and dumping), and automated checks of completion and data validity. As part of the digitisation process, one of the authors (TVV) and a faculty RA trialled four different platforms (i.e., ESRI ‘Survey 123’, Qualtrics, Google Forms, and Microsoft Forms) at a local primary school. The FACTS tool was digitised onto each platform and used by the trained auditors to conduct in-field audits of the same four routes (segments *n* = 28; crossings *n* = 18). The trialling was conducted over four days of the same week, with one platform being trialled per day. Upon completion of the trialling, feedback on the in-field useability of each platform was discussed with the broader project team (MJD and SJC), and the decision to proceed with the ESRI ‘Survey123’ platform [32] was made. This decision was informed by the platform’s user-friendly interface and simple integration of GIS/GPS capabilities due to it being part of the ESRI ArcGIS ecosystem. Although the ESRI ‘Survey123’ platform was selected for use in this study, the FACTS tool can be accessed (available under CC-BY) and digitised onto any desired platform using Additional File 2.

### Scoring protocol

**Fig. 1:**
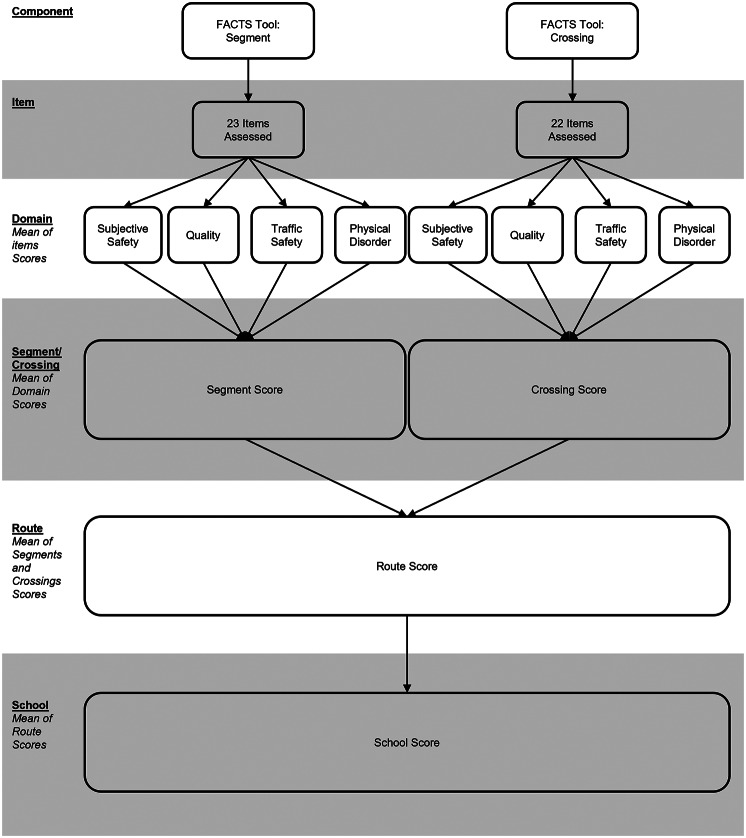
FACTS scoring protocol

The FACTS scoring was designed to be a simple, easy to interpret protocol that could enable program planners to identify problematic SR2S segments/crossings, routes, or overall schools, and provide an understanding of the key issues to enable the targeting of program resources. To do this, a hierarchical scoring protocol consisting of five tiers (i.e., item, domain, segment/crossing, route, and school) was developed (Fig. 1).

Firstly, it was necessary to create and assign the individual segment/crossing component items into theoretically relevant domains. To do this, the authorship team combined their existing knowledge and experience of children’s AST with the diverse body of AST literature. Creation of the provisional domains and assignment of items was guided by theoretical models of children’s active school travel behaviour (e.g., see [33]) and built and social environment factors thought to influence children’s engagement in AST [12, 34]. The process was iterative in nature, beginning at the start of tool development through to after pilot data collection using the tool, and required several brainstorming meetings. Upon completion, the provisional domains and item assignments were put forward to the expert panel for final review and comment. Four domains for both the segment and crossing components were created, covering Subjective Safety, Quality, Traffic Safety, and Physical Disorder (Fig. 1; see Additional File 2).

**Fig. 2:**
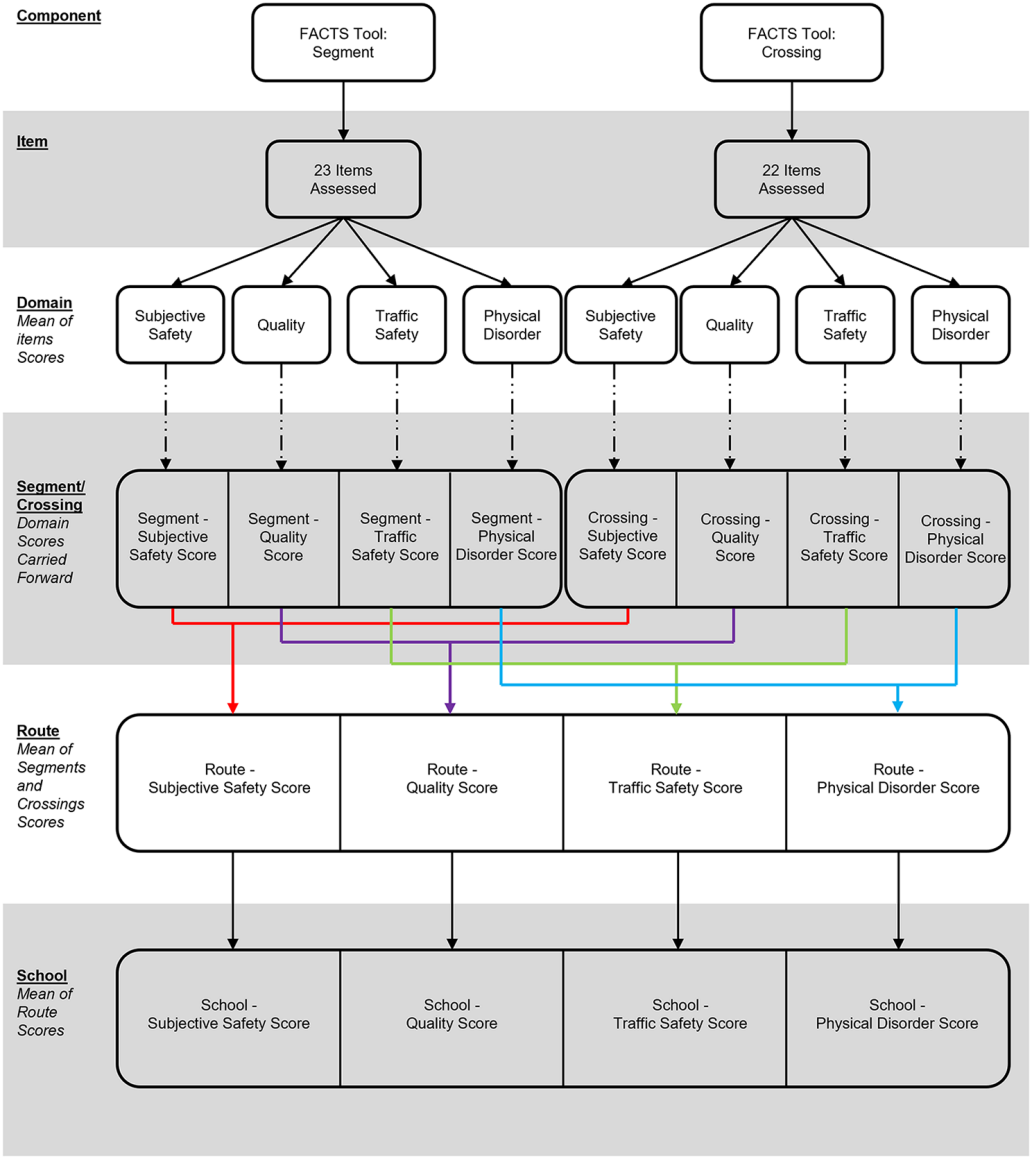
FACTS scoring protocol (cont.)

An overall scoring convention was then developed which provides flexibility in the focus of the scoring, that is, to provide overall summary scores for segments/crossings, routes, and schools, or to provide domain-specific scores at each of these levels (Fig. 1). This flexibility supports querying of the data in different ways to answer different questions of interest. Starting at the smallest unit – the segment or crossing – each domain score is calculated as the mean of items for that domain. From here, summary scores for each segment or crossing can be similarly calculated (i.e., an overall score – i.e., mean - for all domains for the segment or crossing) and summarised for routes and/or schools or, the domain scores can be summarised at the route and/or school level providing route and/or school level domain-specific information (Fig. 2).

All items on the FACTS tool are scored between 0 and 1, with nominal items (i.e., yes/no) being scored as 0/1, and ordinal items scored to range between 0 and 1 (e.g., a little, some, a lot being scored as 0.33, 0.66, 1.00), with the values varying depending on the number of item responses (see Additional File 2). For scoring, items are coded such that the larger values represent an active travel supportive response. Overall, scoring/mean values of ≤ 0.33 were considered poor; >0.33 to ≤ 0.66 were considered moderate; and > 0.66 were considered good. The item scoring was designed this way (i.e., items ranging between 0 and 1 with equal weighting) to ensure ease of use and interpretability amongst end users who are from diverse, nonstatistical backgrounds (e.g., parents, teachers, school principals, SR2S staff, and city planners, among others). This is a deviation from MAPS Global, for example, which uses a far more complex scoring protocol [20, 26, 35].

### Sampling

The primary schools included in the FACTS tool reliability assessment were sampled as part of the broader project’s intended realist evaluation of the Active Streets for Schools program. For the realist evaluation, the sampling frame included primary schools enrolled on the Active Streets for Schools program as part of the 2018–2022 wave of the program *(n* = 52). This study then applied the following sampling approach: from the 52 primary schools enrolled during this period, 23 private and independent primary schools were excluded for not having a priority enrolment area (i.e., catchment). This was done to ensure that most children were enrolled at a school within active travel distance of their home. Six primary schools were also excluded from the sampling frame for being partial primary schools (i.e., Preschool - Year 3) and combined schools (i.e., Preschool – Year 12). This was done to ensure the schools sampled represented only the primary target population of the program (i.e., children in Preschool – Year 6) and to remove concerns with collecting evaluation outcome data at combined schools (e.g., differentiation between primary and secondary school students wearing the same uniform). After exclusions, eight of the 23 remaining public primary schools were then selected, which included four intervention schools (i.e., schools that received implementation of the Active Streets for Schools program) and four matched controls (i.e., schools that had implementation of the Active Streets for Schools program delayed). Intervention schools were purposively selected for their ability to test several realist preliminary program theories developed within the project’s rapid realist review [13]. Control schools were then matched (as similarly as possible) to the intervention schools according to various contextual characteristics (Table 1). School pairings were based on: (1) small population size; (2) large population size; (3) co-location with another primary school (i.e., adjacent to each other); and (4) the number of AST programs enrolled in (single or multiple).

**Table 1:** Characteristics of sampled schools for the reliability assessment

School	Pairing	Year Opened	School Population	ICSEA	# of SR2S	# of AST Programs	Co-located
School A ^I^	1	1977	335	1076	7	2	No
School B ^C^		1978	355	1056	5	2	No
School C ^I^	2	1958	690	1130	5	2	Yes
School D ^C^		1960	795	1139	5	3	Yes
School E ^I^	3	1989	555	1098	4	2	Yes
School F ^C^		1994	510	997	5	2	Yes
School G ^I^	4	1973	452	1079	9	3	No
School H ^C^		1978	418	1070	6	1	No

ICSEA: Index of Community Socio-educational Advantage

^I^Intervention;^C^Control

## Data collection

### Training of auditors

Prior to the data collection, both auditors participated in a two-part training session in March 2021. The first session involved a classroom-based exercise where both auditors reviewed the FACTS tool and accompanying manual to familiarise themselves with item ordering, item definitions and responses (including supporting images), and the interface of the ESRI ‘Survey 123’ app. A formal discussion also took place to provide both auditors the opportunity to raise questions. The second session, conducted on the same day, involved an in-field practical at a local primary school. The practical began with both auditors auditing two of the school’s four SR2S together (segments *n* = 10; crossings *n* = 7), allowing for item responses to be discussed and disagreements resolved. Both auditors then audited the remaining two SR2S separately (segments *n* = 18; crossings *n* = 11), with the agreement between the two auditors being assessed as > 90.0%. Both auditors reported feeling comfortable applying the instrument after completion of the training.

### Audit data to assess reliability

Audit data to assess reliability were collected for the eight sampled schools over a three week period during April 2021. The two trained auditors each conducted audits on the ‘safe routes’ for the same six schools to enable the assessment of inter-rater reliability. For intra-rater reliability, both auditors conducted repeat measures on the ‘safe routes’ for four schools each (eight schools total), which included three different schools per auditor from the six used for inter-rater reliability as well as an additional school each. The mean time between repeat measures on any individual school was three days (SD = 1.2 days, range 2–5) and included a different school audit between measures; this was done to minimise auditor recall bias. All route audits were conducted between 9:00am and 3:00pm so as not to interfere with children travelling to school/commuters; on the rare occasion that children were encountered on the route, all data collection was briefly suspended. Both auditors entered the field with a personal mobile device with a university-issued data sim, the ESRI ‘Survey 123’ app (v 3.11, ESRI, Redlands, CA) installed and the FACTS tool and the school’s SR2S map preloaded. Paper-based versions of the FACTS tool and the school’s SR2S map, as well as a portable power bank, were also carried by both auditors to mitigate against any data loss due to technical failure (e.g., mobile device running out of battery/exceeding daily mobile data allowance).

### Data analysis

All data were extracted from the ESRI platform and imported into R Studio [36] for data preparation and cleaning. The cleaning process was performed to correct entry errors (for IDs of routes/segments/crossings) that occurred during data collection. No other ‘corrections’ were performed. Upon completion, all data were exported into Statistical Package for the Social Sciences v.27 for the reliability analysis [37].

Item-by-item inter-rater and intra-rater reliability were assessed using Cohen’s Kappa statistic for binary/nominal items (8 items per segment component; 12 items per crossing component) and Cohen’s Weighted Kappa statistic for ordinal items (15 items per segment component; 10 items per crossing component). For computation of Cohen’s Weighted Kappa, a linear weighting was applied assuming ordinal item responses were equally distanced [38]. Percentage agreement was also calculated for item-by-item reliability to determine the proportion of instances where both auditors selected the same item response, or instances where the same response was indicated for both the single and repeat measures; percentage agreement above 70% was considered high [19, 39]. The inter- and intra-rater reliability for mean domain, segment/crossing, route, and school scores were assessed using Intraclass Correlation Coefficients (ICCs). ICC values and their 95% confidence intervals were computed based on a single-rater, consistency-agreement, two-way random effects model [40]. Following the guidance for interpreting Kappa values put forth by Landis and Koch [41], values < 0.00 reflected poor agreement, between 0.00 and 0.20 reflected slight agreement, 0.21–0.40 reflected fair agreement, 0.41–0.60 reflected moderate agreement, 0.61–0.80 reflected substantial agreement, and 0.81–1.00 reflected almost perfect agreement. This guidance was also used for interpreting ICC values similar to that of previous reliability research [19].

## Results

### Descriptive characteristics

Table 2 presents the descriptive characteristics for the reliability assessment of the FACTS tool. Overall, there were 438 paired observations collected using the tool’s segment and crossing components, of which 184 were used to assess inter-rater reliability and 254 used to assess intra-rater reliability. The mean completion time for segments was 5:27 min (SD = 5:56) and for crossings was 1:19 min (SD = 1:12).

**Table 2:** Descriptive characteristics of the reliability data

	Inter-rater *n* (%)	Intra-rater *n* (%)
**Paired Observations by Segment Type**		
Street	37 (32.2) ^a^	74 (46.8)
Greenway	78 (67.8) ^a^	84 (53.2)
Total	115 (100.0)	158 (100.0)
**Paired Observations by Crossing Type**		
Street	37 (53.6)	64 (66.6)
Underpass	32 (46.4)	32 (33.3)
Overpass	0 (0.0)	0 (0.0)
Total	69 (100.0)	96 (100.0)

^a^The number of reported greenway and street segments differed between the two auditors due to one disagreement on the filter classification item (S18)

### Reliability assessment

#### Item-by-item reliability

Item-by-item reliability results for the segment and crossing components of the FACTS tool are presented in Table 3. For segment inter-rater reliability, seven of the 23 items (30.4%) had almost perfect agreement, five items (21.7%) had substantial agreement, seven items (30.4%) had moderate agreement, three items (13.0%) had fair agreement, and a Kappa statistic could not be computed for one item (4.3%) due to one of the auditor’s ratings being constant values. Eighteen of the 23 items (78.3%) had high percentage agreement between the two auditors. For intra-rater reliability, six of the 23 segment items (26.1%) had almost perfect agreement, five items (21.7%) had substantial agreement, nine items (39.1%) had moderate agreement, one item (4.3%) had fair agreement, one item (4.3%) had slight agreement, and for one item (4.3%) a Kappa statistic could not be calculated due constant values for one of the measure ratings (i.e., the single or repeat). Percentage agreement between the single and repeat measures was high for 18 of the 23 items (78.3%).

**Table 3:** Item-by-item inter- and intra-rater reliability assessment

Segment		Inter-rater			Intra-rater		
Item number	Item	Percentage Agreement (%)	Kappa (95% CI)	p-value	Percentage Agreement (%)	Kappa (95% CI)	p-value
S1	Describe the predominant path design for this segment:	100.0	1.000	< 0.001	97.5	0.891 (0.751–1.031)	< 0.001
S2	What is the width of the majority of the path?	90.4	0.893 (0.832–0.954)	< 0.001	84.8	0.773 (0.680–0.866)	< 0.001
S3	Are there poorly maintained sections of the path surface that constitute major trip hazards (e.g., cracks, raised sections, misalignments, etc.)?	51.3	0.521 (0.410–0.631)	< 0.001	59.5	0.581 (0.483–0.679)	< 0.001
S4	Are there temporary obstructions on the segment/path (e.g., fallen/overgrown tree branches, shrub overgrowth, building works)?	88.7	0.731 (0.595–0.868)	< 0.001	81.6	0.579 (0.443–0.715)	< 0.001
S5	Are there permanent obstructions on the segment/ path (e.g., bus shelter, street furniture, light post)?	98.3	0.658 (0.213–1.102)	< 0.001	98.1	0.562 (0.117–1.006)	< 0.001
S6	What is the slope of the majority of the segment?	75.7	0.652 (0.533–0.771)	< 0.001	81.0	0.693 (0.590–0.796)	< 0.001
S7	Are there any potentially dangerous sections of the route/segment/path (e.g., steep section, steep cross-slope, blind corner, note: inherent, not due to poor maintenance)?	87.8	0.240 (-0.032–0.511)	0.007	94.3	0.441 (0.136–0.745)	< 0.001
S8	How much of the length of the route/segment/path is shaded (e.g., trees or awnings)?	65.2	0.476 (0.347–0.604)	< 0.001	62.0	0.421 (0.299–0.544)	< 0.001
S9	Overall condition of most buildings and houses (if any)?	73.0	0.532 (0.373–0.691)	< 0.001	70.9	0.569 (0.443–0.695)	< 0.001
S10	Overall condition of dwelling associated gardens/fences (if any)?	65.2	0.584 (0.454–0.714)	< 0.001	67.7	0.619 (0.509–0.728)	< 0.001
S11	Overall condition of public greenspace areas (e.g., the verge, median strip, greenspace, not dwelling associated gardens)?	64.3	0.508 (0.384–0.631)	< 0.001	66.5	0.583 (0.480–0.686)	< 0.001
S12	Is graffiti/tagging (not murals) present?	79.1	0.743 (0.649–0.838)	< 0.001	77.2	0.724 (0.646–0.803)	< 0.001
S13	Is there litter present?	62.6	0.353 (0.212–0.494)	< 0.001	63.3	0.350 (0.222–0.477)	< 0.001
S14	Is there evidence of dumping present (e.g., furniture, electrical goods, broken bikes, cars/car-parts, etc.)?	96.5	0.799 (0.609–0.990)	< 0.001	93.0	0.584 (0.363–0.804)	< 0.001
S15	Are any other signs of disorder present (e.g., broken/boarded windows, abandoned buildings, bottles/broken glass, drug paraphernalia)?	97.4	-^a^	-^a^	98.7	-^a^	-^a^
S16	Does the segment feel safe for use by children? *Account for physical condition*,* visibility*,* and indicators of social disorder*.	97.4	0.387 (-0.162–0.937)	< 0.001	95.6	0.200 (-0.168–0.568)	0.012
S17	Are there informal (i.e., unpaved, ‘goat track’ type) paths that link to the segment/path?	89.6	0.442 (0.178–0.706)	< 0.001	91.8	0.475 (0.233–0.717)	< 0.001
S18	Does this path/segment go through a greenbelt/greenway or is it adjacent to a street?	99.1	0.980 (0.942–1.019)	< 0.001	100.0	1.000	< 0.001
S19	Road-side buffer: i.e., typical path distance from road (i.e., width of road-side buffer), OR (where no path), typical distance from the route to the road:	97.3	0.937 (0.815–1.059)	< 0.001	100.0	1.000	< 0.001
S20	How many traffic lanes are present (include traffic and turning lanes, choose predominant)?	97.3	0.883 (0.640–1.126)	< 0.001	98.6	0.907 (0.715–1.099)	< 0.001
S21	How many driveways are there? *Do not include pedestrian-only access*.	94.6	0.957 (0.898–1.016)	< 0.001	93.2	0.946 (0.900–0.992)	< 0.001
S22	Posted speed limit (majority of the segment):	100	1.000	< 0.001	100.0	1.000	< 0.001
S23	Are traffic-calming characteristics (e.g., speed humps/cushions/tables, chicanes, rumble bars, bollards, altered colouration, signage [e.g., school zone, pedestrian zone], or road narrowing kerb extensions) present along the segment?	81.1	0.415 (0.056–0.774)	0.011	87.8	0.663 (0.461–0.866)	< 0.001
	Crossing	Inter-rater			Intra-rater		
Item number	Item	Percentage Agreement (%)	Kappa (95% CI)	P-value	Percentage Agreement (%)	Kappa (95% CI)	P-value
C1	Does this crossing take place on a pedestrian overpass, underpass or bridge?	100.0	1.000	< 0.001	100.0	1.000	< 0.001
C2	Is there adequate daytime (natural or artificial) lighting for use of the underpass during regular (daytime) school hours?	100.0	-^b^	-^b^	100.0	-^b^	-^b^
C3	Is graffiti/tagging (not murals) present?	87.5	0.848 (0.699–0.997)	< 0.001	90.6	0.887 (0.764–1.009	< 0.001
C4	Is litter present?	68.8	0.380 (0.071–0.688)	0.005	65.6	0.487 (0.250–0.724)	< 0.001
C5	Is there evidence of dumping (e.g., furniture, electrical goods, broken bikes, cars/car-parts, etc.) present?	100.0	-^b^	-^b^	100.0	-^b^	-^b^
C6	Are any other signs of disorder (e.g., bottles/broken glass, drug paraphernalia present?	96.9	0.652 (0.023–1.281)	< 0.001	93.8	0.467 (-0.160–1.094)	0.008
C7	Does the underpass/overpass/bridge feel safe for use by children? *Account for physical condition*,* visibility*,* and indicators of social disorder.*	100.0	-^b^	-^b^	100.0	-^b^	-^b^
C8	What is the width of the majority of the path/walkway?	100.0	1.000	< 0.001	100.0	1.000	< 0.001
C9	Are there poorly maintained sections of the path/walkway that constitute major trip hazards (e.g., cracks, raised sections, misalignment, etc.)?	75.0	0.143 (-0.088–0.374)	0.078	87.5	0.439 (0.059–0.819)	< 0.001
C10	Are stairs used to enter/exit the underpass/overpass/bridge?	100.0	-^b^	-^b^	100.0	-^b^	-^b^
C11	What is the slope of the majority of the underpass/overpass/bridge?	100.0	1.000	< 0.001	100.0	1.000	< 0.001
C12	Are there any potentially dangerous sections of the underpass/overpass/bridge (e.g., steep section, steep cross-slope, blind corner, note: inherent, not due to poor maintenance)?	100.0	-^a^	-^a^	100.0	-^a^	-^a^
C13	Road complexity at crossing?	94.6	0.892 (0.746–1.038)	< 0.001	92.2	0.897 (0.808–0.986)	< 0.001
C14a	Crossing control: Are there traffic signals or is it a supervised school crossing?	97.3	0.893 (0.688–1.098)	< 0.001	100.0	1.000	< 0.001
C14b	If no traffic signals/supervised crossing, are there give way signs, stop signs and/or a roundabout?	90.6	0.529 (0.082–0.977)	< 0.001	92.3	0.769 (0.554–0.984)	< 0.001
C15	Are traffic-calming characteristics (e.g., speed humps/ cushions/tables, chicanes, rumble bars, bollards, altered colouration, signage, or road narrowing kerb extensions) present nearby?	86.5	0.485 (0.125–0.845)	< 0.001	87.5	0.487 (0.186–0.788)	< 0.001
C16	How many traffic lanes are crossed? *Include bus and turning lanes but not cycling lanes*.	100.0	-^b^	-^b^	98.4	0.855 (0.551–1.158)	< 0.001
C17	Crosswalk treatment: does this crossing have high visibility striping/zebra crossing, different material than the road, and/or a raised crosswalk? (*at crossing only*).	97.3	0.843 (0.542–1.143)	< 0.001	96.9	0.840 (0.625–1.055)	< 0.001
C18	Is a protected refuge island present?	100.0	1.000	< 0.001	95.3	0.815 (0.612–1.017)	< 0.001
C19	Crossing kerbs:	94.6	0.784 (0.520–1.048)	< 0.001	100.0	1.000	< 0.001
C20	Are there other potential crossing issues (e.g., poor road surface, potholes)?	70.3	0.181 (-0.147–0.509)	0.235	82.8	0.423 (0.145–0.701)	< 0.001
C21	Does the crossing feel safe for use by children (consider number of lanes, traffic calming characteristics, signalisation etc.)?	81.1	0.367 (0.014–0.720)	0.012	87.5	0.430 (0.103–0.757)	< 0.001

^a^Could not be computed due to one auditor’s ratings or single/repeat measure being constant values.

^b^Could not be computed due to both auditor’s ratings or single and repeat measures being constant values

For inter-rater reliability of the crossing component, eight of the 22 items (36.4%) had almost perfect agreement, two items (9.1%) had substantial agreement, two items (9.1%) had moderate agreement, two items (9.1%) had fair agreement, two items (9.1%) had slight agreement, and Kappa values could not be calculated for six items (27.3%) due to constant values for either one or both auditor’s ratings. Twenty one out of the 22 crossing component items (95.6%) had high percentage agreement between both auditors. For intra-rater reliability, 10 items (45.5%) had almost perfect agreement, one item (4.5%) had substantial agreement, six items (27.3%) had moderate agreement, and Kappa values could not be calculated for five of the 23 items (22.7%) due to one or both single and repeat measure ratings being constant values. Percentage agreement between the single and repeat measures was high for 21 of the 22 crossing items (95.6%).

### Domain scores reliability

Inter- and intra-rater reliability results for the FACTS tool segment and crossing domains scores are presented in Table 4. For inter-rater reliability of segment domain scores, the Subjective Safety domain (ICC = 0.388), which was based on a single binary item with a percentage agreement of 97.4%, had fair agreement, the Quality and Traffic Safety domains (ICC = 0.834 and 0.975 respectively) had almost perfect agreement, and the Physical Disorder domain (ICC = 0.663) had substantial agreement between the two auditor’s domain scores. For intra-rater reliability, the Subjective Safety domain (ICC = 0.200) had slight agreement, the Quality and Physical Disorder domains (ICC = 0.691 and 0.698 respectively) had substantial agreement, and the Traffic Safety domain (ICC = 0.949) had almost perfect agreement between the single and repeat measure domain scores.

**Table 4:** Reliability of domain, segment/crossing, route, and school scores

		Inter-rater			Intra-rater		
Domain Scores	*n* of items	Mean (SD)	ICC (95% CI)	p-value	Mean (SD)	ICC (95% CI)	p-value
Segment							
*Subjective Safety*	1^a^	0.98 (0.15)	0.388 (0.221–0.533)	< 0.001	0.97 (0.17)	0.200 (0.046–0.345)	0.006
*Quality*	8^a^	0.66 (0.20)	0.834 (0.769–0.882)	< 0.001	0.68 (0.19)	0.691 (0.600–0.765)	< 0.001
*Traffic Safety*	6^a^	0.84 (0.25)	0.975 (0.965–0.983)	< 0.001	0.77 (0.27)	0.949 (0.930–0.962)	< 0.001
*Physical Disorder*	7^a^	0.70 (0.10)	0.663 (0.547–0.754)	< 0.001	0.72 (0.10)	0.698 (0.608–0.770)	< 0.001
Crossing							
*Subjective Safety*	2	0.91 (0.29)	0.427 (0.214–0.602)	< 0.001	0.92 (0.28)	0.457 (0.283–0.601)	< 0.001
*Quality*	8	0.90 (0.21)	0.555 (0.368–0.699)	< 0.001	0.90 (0.22)	0.691 (0.571–0.783)	< 0.001
*Traffic Safety*	8	0.62 (0.37)	0.991 (0.986–0.995)	< 0.001	0.54 (0.35)	0.985 (0.978–0.990)	< 0.001
*Physical Disorder*	4	0.87 (0.10)	0.720 (0.500–0.853)	< 0.001	0.88 (0.11)	0.592 (0.311–0.777)	< 0.001
Segment Scores	-	0.80 (0.11)	0.878 (0.829–0.914)	< 0.001	0.78 (0.11)	0.790 (0.723–0.842)	< 0.001
Crossing Scores	-	0.81 (0.19)	0.798 (0.693–0.870)	< 0.001	0.79 (0.18)	0.803 (0.719–0.864)	< 0.001
Route Scores	-	0.82 (0.07)	0.866 (0.753–0.929)	< 0.001	0.81 (0.07)	0.829 (0.709–0.902)	< 0.001
School Scores	-	0.83 (0.03)	0.850 (0.266–0.978)	0.008	0.81 (0.04)	0.851 (0.425–0.968)	0.002

^a^Total n = 22 as question (S17) is not included in the scoring protocol

For inter-rater reliability of crossing domain scores, the Subjective Safety and Quality domains (ICC = 0.427 and 0.555 respectively) had moderate agreement, the Traffic Safety domain (ICC = 0.991) had almost perfect agreement, and the Physical Disorder domain (ICC = 0.720) had substantial agreement between the two auditor’s domain scores. For intra-rater reliability, the Subjective Safety and Physical Disorder domains (ICC = 0.457 and 0.592 respectively) had moderate agreement, the Quality domain (ICC = 0.691) had substantial agreement, and the Traffic Safety domain (ICC = 0.985) had almost perfect agreement between the single and repeat measure domain scores.

For inter-rater reliability of the remaining scoring, segment scores (ICC = 0.878) had almost perfect agreement, crossing scores (ICC = 0.798) had substantial agreement, and both route and school scores (ICC = 0.866 and 0.850 respectively) had almost perfect agreement between the two auditor’s ratings. For intra-rater reliability, segment and crossing scores (ICC = 0.790 and 0.803 respectively) had substantial agreement, and route and school scores (ICC = 0.829 and 0.851 respectively) had almost perfect agreement between the single and repeat measure ratings.

## Discussion

The present study described the reliability assessment of the FACTS tool for characterising the microscale built environment of promoted ‘safe routes’ for children’s AST within SR2S programs. For item-by-item inter- and intra-rater reliability, 31 (68.9%) and 37 (82.2%) of the 45 items had moderate to almost perfect agreement respectively, seven (15.6%) and two (4.4%) of the 45 items had below moderate agreement respectively, and a Kappa statistic could not be calculated for seven (15.6%) and six (13.3%) of the 45 items respectively, due to constant values, though all these items had percentage agreement > 95.0%. For inter- and intra-rater reliability of the segment domain scores, three of the four domains had substantial to almost perfect agreement. For inter- and intra-rater reliability of the crossing domain scores, all four domains had moderate to almost perfect agreement. For inter- and intra-rater reliability of the segment, crossing, route, and school scores, all had substantial to perfect agreement. Overall, these results indicate the FACTS tool is reliable and fit for its purpose of assessing the microscale built environment of promoted ‘safe routes’ for children’s AST within SR2S programs.

When considered overall, the FACTS tool had good reliability for assessing the microscale built environment of promoted ‘safe routes’ for children’s AST within SR2S programs. Though item-by-item inter-rater reliability appears lower than previous field audit tools [20, 24, 26, 27], with only 68.9% of FACTS tool items in the moderate to almost perfect range, this is due to the inability to calculate Kappa statistics for seven items (15.6%) due to constant values (Table 3). Using percentage agreement to categorise these seven items, (an approach previously used in cases of low Kappa scores due to insufficient variability [24, 26]), > 84.0% of the FACTS tool’s items are in the moderate to almost perfect range, more in line with existing field audit tools. For the small number of audit tools that have had their intra-rater reliability assessed, the FACTS tool reported similar levels of intra-rater reliability, with between 80.0% and 90.0% of items reported within the moderate to almost perfect range [24, 25]. Moreover, many microscale field audit tools use a combination of objective and subjective judgement items that have displayed varying levels of reliability, with the subjective items typically displaying lower levels [18–20, 24–26]. Consistent with the findings of previous research, the subjective items on the FACTS tool (e.g., presence of litter) demonstrated the lowest level of reliability, with the more objective items (e.g., path width) having reported higher levels (Table 3). Placing greater emphasis on subjective items during training sessions and within supplementary materials (e.g., training manuals) may aid improvement during future use. However, given the nature of subjective judgement items, this is likely to be an ongoing challenge facing field audit tools such as the FACTS tool. In addition, there is likely a threshold beyond which additional training provides no benefit, as the subjective items are likely to (truly) vary more than objectively measured items.

Typically, in SR2S programs, only a singular infrastructure change is made per school. This is due to the limited funding typically made available, the substantial financial cost associated with implementing built environmental infrastructure changes (e.g., children’s crossing) [42], and in the specific case assessed here, to the program’s inclusive enrolment policies resulting in the enrolment of a large number of schools. Consequently, program planners are required to make judgements on which ‘safe route’, and which point along this route, will benefit most from an infrastructure improvement. Without on-the-ground information, this can pose a significant challenge. The FACTS tool can not only assist program planners in identifying problematic segments/crossings along the ‘safe routes’ for priority consideration, but, through the domain summaries, can also indicate what type of change may be of highest priority. For example, a segment that scored low in the Quality domain may suggest relevelling of the pavement surface to be a priority. Additionally, by allowing summaries from the segment/crossing level to the school level, the FACTS tool could be used to assess a school’s eligibility for receiving infrastructure changes on a “needs” basis [13]. This approach would allow infrastructure funding to be allocated to a smaller number of schools that are most in need of improvements. This more targeted approach could enable larger (more costly) infrastructure changes on routes most in need, or allow for multiple infrastructure changes to be made at a given school, improving multiple of the ‘safe routes’ (or multiple sections of one particularly problematic route), which will likely have greater impact on children’s AST behaviour.

The FACTS tool was developed specifically for assessing the microscale built environment of promoted ‘safe routes’ for children’s AST within SR2S programs. In doing so, it was possible to create a streamlined tool consisting of only 45 items. This places the FACTS tool at the lower end of the item inclusion range, with existing tools containing between 15 and 123 items [18–20, 23–28]. This results in the FACTS tool having a relatively short time for completion, without compromising on the comprehensiveness of the information collected [27]. This is particularly important given the large number of schools that often enrol in SR2S programs (e.g., 81 primary schools out of 103 in Canberra). That said, it is acknowledged that although the FACTS tool has a relatively short completion time, this does not account for auditor travel time to school sites, and would not mitigate the high cost of in-field audits to the same extent as Google Street View adaptations [19, 28]. However, Google Street View does not always provide up to date imagery, nor complete coverage. For example, off-street footpaths, which are critical for SR2S programs due to most ‘safe routes’ using off-street footpaths (thus avoiding traffic safety concerns) may not have imagery. Thus, the FACTS tool is highly suitable for informing and/or evaluating SR2S programs and program planners and evaluators of future SR2S programs should consider its use.

This study and the FACTS tool have several strengths. To the best of our knowledge, the FACTS tool is the first microscale audit tool designed specifically for assessing the built environment of identified and promoted ‘safe routes’ for children’s AST within SR2S programs in Australia. It is a practical and reliable tool that can be used for informing and evaluating SR2S programs. Additionally, it extends the suite of available microscale audit tools to a new context/environment. Throughout the development and refinement stages of the FACTS tool, there was engagement with stakeholders, including a multidisciplinary expert panel and the project’s industry partner, who was actively engaged in SR2S program implementation. This has ensured the FACTS tool content is conceptually relevant for assessing the microscale built environment of ‘safe routes’ for children’s AST, but also provides useful information for program planners and evaluators implementing and/or evaluating these types of AST programs. Observers using the FACTS tool were able to collect reliable microscale built environment information with minimal training, suggesting the FACTS tool is easy to use.

However, both the study and the FACTS tool are not without limitations. All data used for the reliability assessment was collected within the suburban environment of one Australian city; thus, the application and reliability of the FACTS tool in rural and/or different geographic contexts may be limited, and further assessment in these contexts would be advisable. Due to the logic of the ‘safe routes’ (i.e., routes that avoid main roads and crossings), the reliability of the crossing component was based on a smaller sample size, which has resulted in broader confidence intervals. A larger study including more schools/routes, or a study that selects specific routes with certain characteristics (e.g., routes that contain overpasses), would enable further reliability assessment of FACTS tool components that had limited responses in this study. These approaches were not practical for this study, as the schools/routes audited were tied to a program and its evaluation. Commonly used methods for scale/tool development, such as factor analysis and measures of internal consistency (e.g., Cronbach’s alpha), were not used in this study. This decision, however, was made because these approaches have previously been identified as unsuitable for scales/tools designed for assessing environments, as conceptually related features may not co-exist [20].

## Conclusions

The FACTS tool provides SR2S program planners and evaluators with a useful and reliable tool for characterising the microscale built environment of promoted ‘safe routes’ for children’s AST within SR2S programs. The information collected can be used to inform selection of built environment infrastructure changes, as well as evaluate program effectiveness. Overall, the FACTS tool should be considered for use by SR2S program planners within the suburban Australian context. Further testing will likely be required for broader geographic use.

## Electronic supplementary material

Below is the link to the electronic supplementary material.

Supplementary Material 1

Supplementary Material 2

## Data Availability

The datasets generated and/or analysed during the current study are available in the Digital Commons Data institutional data repository, https://data.mendeley.com/datasets/89gdrczzdd/1.

## Abbreviations

AST: Active School Travel
FACTS: Field Audit for Children’s Active Transport to School
ICC: Intraclass Correlation Coefficient
MAPS: Microscale Audit of Pedestrian Streetscapes
RAs: Research Assistants
SR2S: Safe Routes to School
