# Supplementary material for: Reliability assessment of the ‘field audit for children’s active transport routes to school’ (FACTS) tool
Source: BMC Public Health. 2024 Oct 14;24:2812. doi: 10.1186/s12889-024-20285-3 (PMC11472530; doi:10.1186/s12889-024-20285-3)
Supplement: Supplementary file 1 — Supplementary Material 1 [file 12889_2024_20285_MOESM1_ESM.pdf]

Additional File 1: FACTS Tool Interview Guide

Welcome and thank you for agreeing to participate in this interview. This interview is designed to assess your current thoughts and experiences as a user of the FACTS tool. These questions are specific to the questions on the segment and crossing forms only. This interview will take between 30-60 minutes. All information provided during this interview will remain strictly confidential. Do have any questions for me before we begin? Are you ready to proceed?

1. You were involved with the Active Streets route audits, could you share your experiences of using the FACTS tool with me?
    i. How do you feel about the useability?
    ii. How do you feel about the structure of questions on the FACTS tool?

2. From a user's perspective, what do you consider to be the strengths of the FACTS tool?
    i. That's interesting, could you explain why you consider that a strength?

3. What do you consider to be the weaknesses of the FACTS tool?
    i. That's a useful point, could you expand on that further?

4. Are there any other comments to be shared about the strengths and weaknesses of the FACTS tool before we move on?

5. What are your feelings towards the current set of questions on the FACTS tool?
    i. Do you feel there is a need for modification to the current questions?

6. Are there any questions you think should be added or removed from the FACTS tool?
    i. Why do you think that would be a useful addition/removal?
    ii. How would you feel about a question involving stranger danger?
    iii. Question involving natural debris?
    iv. Question involving animal presence/excrement?
    v. Question for on-segment/on-crossing recommendations?

7. How would you feel about the addition or modification of questions to allow for more of a qualitative (detailed) response?
    i. What are your opinions about the usefulness of this addition for an auditor?
    ii. From a user's perspective, are there any issues with this addition you can foresee?

8. What do you think about the digitisation of the FACTS tool?
    i. In what format would you like to see the digitised tool?
    ii. What features would you like to see on the digitised tool?
    iii. What are your thoughts on geotagging images of issues while on audit?
    iv. Do you have any concerns with using a digitised version of the FACTS tool?

9. Are there any other comments or opinions you would like to share about your experiences with using the FACTS tool?

That concludes the interview. Thank you very much for your time today.
