# Supplementary material for: Reliability assessment of the ‘field audit for children’s active transport routes to school’ (FACTS) tool
Source: BMC Public Health. 2024 Oct 14;24:2812. doi: 10.1186/s12889-024-20285-3 (PMC11472530; doi:10.1186/s12889-024-20285-3)
Supplement: Supplementary file 2 — Supplementary Material 2 [file 12889_2024_20285_MOESM2_ESM.pdf]

Additional File 2: FACTS Tool and Scoring

Segment Component:

Item number	Item	Response	Scoring value	Subdomain	Domain
S1	Describe the predominant path design for this segment:	Paved path present and continuous	1	Quality – Design	Quality
		Paved path present but not continuous	0.8		
		Unpaved path (i.e., gravel path) present and continuous	0.6		
		Unpaved path present but not continuous	0.4		
		No formal path, but informal track (or 'goat' track)	0.2		
		No path or informal track	0		
S2	What is the width of the majority of the path?	>2m	1	Quality – Design	Quality
		1.5m-2m	0.75		
		1-1.49m	0.5		
		<1m	0.25		
		N/A (no path)	0		
S3	Are there poorly maintained sections of the path surface that constitute major trip hazards (e.g., cracks, raised sections, misalignments, etc.)?	None	1	Quality – Maintenance	Quality
		One	0.66		
		A Few	0.33		
		Many	0		
		N/A (no path)	0		
S4	Are there temporary obstructions on the segment/path (e.g., fallen/overgrown tree branches, shrub overgrowth, building works)?	Yes	0	Quality – Maintenance	Quality
		No	1		
S5	Are there permanent obstructions on the segment/ path (e.g., bus shelter, street furniture, light post)?	Yes	0	Quality – Design	Quality
		No	1		
S6	What is the slope of the majority of the segment?	Flat or gentle	1	Quality – Design	Quality
		Moderate	0.5		
		Steep	0		
S7		Yes	0	Quality – Design	Quality

	Are there any potentially dangerous sections of the route/segment/path (e.g., steep section, steep cross-slope, blind corner, note: inherent, not due to poor maintenance)?	No	1		
S8	How much of the length of the route/segment/path is shaded (e.g., trees or awnings)?	76-100%	1	Quality – Design	Quality
		51-75%	0.75		
		26-50%	0.5		
		1-25%	0.25		
		No coverage	0		
S9	Overall condition of most buildings and houses (if any)?	Excellent	1	N/A	Physical Disorder
		Good	0.66		
		Fair	0.33		
		Poor	0		
		No buildings	N/A		
S10	Overall condition of dwelling associated gardens/fences (if any)?	Excellent	1	N/A	Physical Disorder
		Good	0.66		
		Fair	0.33		
		Poor	0		
		No visible gardens/fences	N/A		
S11	Overall condition of public greenspace areas (e.g., the verge, median strip, greenspace, not dwelling associated gardens)?	Excellent	1	N/A	Physical Disorder
		Good	0.66		
		Fair	0.33		
		Poor	0		
		No public greenspace	N/A		
S12	Is graffiti/tagging (not murals) present?	None	1	N/A	Physical Disorder
		A little (present)	0.66		
		Some (very noticeable)	0.33		
		A lot (overwhelming)	0		
S13	Is there litter present?	None	1	N/A	Physical Disorder
		A little (present)	0.66		
		Some (very noticeable)	0.33		
		A lot (overwhelming)	0		
S14		None	1	N/A	Physical Disorder
		A little (present)	0.66		

	Is there evidence of dumping present (e.g., furniture, electrical goods, broken bikes, cars/car-parts, etc)?	Some (very noticeable)	0.33		
		A lot (overwhelming)	0		
S15	Are any other signs of disorder present (e.g., broken/boarded windows, abandoned buildings, bottles/broken glass, drug paraphernalia)?	Yes	0	N/A	Physical Disorder
		No	1		
S16	Does the segment feel safe for use by children? *Account for physical condition, visibility, and indicators of social disorder*	Yes	1	N/A	Subjective Safety
		No	0		
S17	Are there informal (i.e., unpaved, 'goat track' type) paths that link to the segment/path?	Yes	N/A	N/A	N/A
		No	N/A		
S18	Does this path/segment go through a greenbelt/greenway or is it adjacent to a street?	Yes (end form here)	1	N/A	Traffic Safety
		No (continue to S19)	N/A[a]		
S19	Road-side buffer: i.e., typical path distance from road (i.e., width of road-side buffer), OR (where no path), typical distance from the route to the road:	3+m from kerb	1	N/A	Traffic Safety
		2 - <3m from kerb	0.75		
		1 - <2m from kerb	0.5		
		≤1m from kerb	0.25		
		Next to kerb	0		
S20	How many traffic lanes are present (include traffic and turning lanes, choose predominant)?	1	1	N/A	Traffic Safety
		2	0.75		
		3	0.5		
		4	0.25		
		5 or more	0		
S21	How many driveways are there? *Do not include pedestrian-only access*	None	1	N/A	Traffic Safety
		1-2	0.66		
		3-5	0.33		
		6+	0		
S22	Posted speed limit (majority of the segment):	≤10km	1	N/A	Traffic Safety
		11-25km	0.8		
		26-40km	0.6		
		41-60km/None posted	0.4		
		61-80km	0.2		

		>80km	0		
S23	Are traffic-calming characteristics (e.g., speed humps/cushions/tables, chicanes, rumble bars, bollards, altered colouration, signage [e.g., school zone, pedestrian zone], or road narrowing kerb extensions) present along the segment?	Yes	1	N/A	Traffic Safety
		No	0		

Subjective Safety Domain – S16.

Quality Domain – Design score: Mean of S1, S4, S5, S6, S7, and S8; Maintenance score: Mean of S2 and S3; Mean of Design score and Maintenance score.

Traffic Safety Domain – S18 only (if greenway segment) **OR** mean of S19, S20, S21, S22, and S23 (if street segment)[a].

Physical Disorder Domain – Mean of S9, S10, S11, S12, S13, S14, and S15.

Crossing Component:

Item number	Item	Response	Scoring value	Subdomain	Domain
C1	Does this crossing take place on a pedestrian overpass, underpass or bridge?	Yes, underpass (continue to C2)	1	N/A	Traffic Safety
		Yes, overpass or bridge (skip to C3)	1		
		No, Street crossing (skip to C13)	N/A[a]		
C2	Is there adequate daytime (natural or artificial) lighting for use of the underpass during regular (daytime) school hours?	Yes	1	Quality – Design	Quality
		No	0		
C3	Is graffiti/tagging (not murals) present?	None	1	N/A	Physical Disorder
		A little (present)	0.66		
		Some (very noticeable)	0.33		
		A lot (overwhelming)	0		
C4	Is litter present?	None	1	N/A	Physical Disorder
		A little (present)	0.66		
		Some (very noticeable)	0.33		
		A lot (overwhelming)	0		
C5	Is there evidence of dumping (e.g., furniture, electrical goods, broken bikes, cars/car-parts, etc) present?	None	1	N/A	Physical Disorder
		A little (present)	0.66		
		Some (very noticeable)	0.33		
		A lot (overwhelming)	0		
C6	Are any other signs of disorder (e.g., bottles/broken glass, drug paraphernalia present?	Yes	0	N/A	Physical Disorder
		No	1		
C7	Does the underpass/overpass/bridge feel safe for use by children? *Account for physical condition, visibility, and indicators of social disorder*	Yes	1	N/A	Subjective Safety
		No	0		
C8	What is the width of the majority of the path/walkway?	>2m	1	Quality – Design	Quality
		1.5-2m	0.66		
		1-1-49m	0.33		
		<1m	0		

C9	Are there poorly maintained sections of the path/walkway that constitute major trip hazards (e.g., cracks, raised sections, misalignment, etc)?	None	1	Quality – Maintenance	Quality
		One	0.66		
		A Few	0.33		
		Many	0		
C10	Are stairs used to enter/exit the underpass/overpass/bridge?	No stairs/Stairs one end with ramp/Stairs both ends with ramps both ends	1	Quality – Access	Quality
		Stairs one end (no ramp)/Stairs both ends, ramp option one end	0.5		
		Stairs both ends (no ramps)	1		
C11	What is the slope of the majority of the underpass/overpass/bridge?	Flat or gentle	1	Quality – Design	Quality
		Moderate	0.5		
		Steep	0		
C12	Are there any potentially dangerous sections of the underpass/overpass/bridge (e.g., steep section, steep cross-slope, blind corner, note: inherent, not due to poor maintenance)?	Yes	0	Quality – Design	Quality
		No	1		
C13	Road complexity at crossing?	Crossing of a one-way street	1	N/A	Traffic Safety
		Crossing of a two-way street	0.66		
		T-junction	0.33		
		≥4-way intersection	0		
C14a	Crossing control: Are there traffic signals or is it a supervised school crossing?	Yes (skip to C15)	1	N/A	Traffic Safety
		No (continue to C14b)	N/A[a]		
C14b	If no traffic signals/supervised crossing, are there give way signs, stop signs and/or a roundabout?	Yes	0.5	N/A	Traffic Safety
		No	0		
C15	Are traffic-calming characteristics (e.g., speed humps/ cushions/tables, chicanes, rumble bars, bollards, altered colouration, signage, or road narrowing kerb extensions) present nearby?	Yes	1	N/A	Traffic Safety
		No	0		
C16	How many traffic lanes are crossed? *Include bus and turning lanes but not cycling lanes*	1	1	N/A	Traffic Safety
		2	0.75		
		3	0.5		

		4	0.25		
		5 or more	0		
C17	Crosswalk treatment: does this crossing have high visibility striping/zebra crossing, different material than the road, and/or a raised crosswalk? (at crossing only)	Yes	1	N/A	Traffic Safety
		No	0		
C18	Is a protected refuge island present?	Yes	1	N/A	Traffic Safety
		No	0		
C19	Crossing kerbs:	Ramps/kerb cuts line up with crossing	1	Quality – Access	Quality
		Ramps/kerb cuts do not line up with crossing	0.5		
		Ramp/kerb cut on one side only/No ramps/kerb cuts	0		
C20	Are there other potential crossing issues (e.g., poor road surface, potholes)?	Yes	0	Quality – Maintenance	Quality
		No	1		
C21	Does the crossing feel safe for use by children (consider number of lanes, traffic calming characteristics, signalisation etc)?	Yes	1	N/A	Subjective Safety
		No	0		

Subjective Safety Domain – C7 (if underpass, overpass, or bridge) **OR** C22 (if street crossing).

Quality Domain – Design: C2, C8, C11 and C12 (if underpass); C8, C11, and C12 (if overpass/bridge) **OR** N/A (if street crossing); Maintenance: C9 (if underpass, overpass, or bridge) **OR** C20 (if street crossing); Access: C10 (if underpass, overpass, or bridge) **OR** C19 (if street crossing); Mean of Design score, Maintenance score, and Access Score.

Traffic Safety Domain – C1 only (if underpass, overpass, or bridge) **OR** mean of C13, C14a (if traffic signals/supervised crossing) **OR** C14b (if no traffic signals/supervised crossing)[a], C15, C16, C17, and C18.

Physical Disorder Domain – Mean of C3, C4, C5, and C6 **OR** N/A (if street crossing).
